# Are Social Status and Migration Background Associated with Utilization of Non-medical Antenatal Care? Analyses from Two German Studies

**DOI:** 10.1007/s10995-020-02937-z

**Published:** 2020-05-09

**Authors:** Angelique Ludwig, Céline Miani, Jürgen Breckenkamp, Odile Sauzet, Theda Borde, Ina-Merle Doyle, Silke Brenne, Chantal Höller-Holtrichter, Matthias David, Jacob Spallek, Oliver Razum

**Affiliations:** 1 Department of Epidemiology & International Public Health, School of Public Health, Bielefeld University, Bielefeld, Germany; 2 Center for Innovation in Health Economics (ZIG OWL), Bielefeld, Germany; 3 Centre for Statistics, Bielefeld University, Bielefeld, Germany; 4 Alice Salomon Hochschule Berlin, University of Applied Sciences, Berlin, Germany; 5 Institute for General Practice, Hannover Medical School, Hannover, Germany; 6 Institute of General Practice, Medical Faculty, Otto-Von-Guericke-University, Magdeburg, Germany; 7 Clinic for Gynaecology, Charité University Medicine Berlin, Campus Virchow-Klinikum, Berlin, Germany; 8 Department of Public Health, Brandenburg University of Technology, Senftenberg, Germany

**Keywords:** Non-medical antenatal care, Migration background, Education, Socioeconomic status, Pregnancy

## Abstract

**Objective:**

Non-medical antenatal care (ANC) refers to a range of non-medical services available to women during pregnancy aiming at supporting women and prepare them for the birth and the postpartum period. In Germany, they include antenatal classes, breastfeeding classes and pregnancy-specific yoga or gymnastics courses. Studies suggest that various types of non-medical ANC carry benefits for both the women and their babies. Little is known about the uptake of non-medical ANC among different socioeconomic population subgroups, but one may expect lower utilization among socio-economically disadvantaged women. We analyzed factors contributing to the utilization of non-medical ANC in general and antenatal classes in particular.

**Methods:**

Baseline data of the Bielefeld BaBi birth cohort (2013–2016) and the Berlin perinatal study (2011–2012) were analyzed. Comparing the two cohorts allowed to increase the socio-economic and migration background variance of the study population and to capture the effect of the local context on uptake of services. Multivariate logistic regression analyses were performed to study associations between the uptake of non-medical ANC and socio-economic and migration status.

**Results:**

In Berlin and Bielefeld, being a first generation migrant and having lower levels of education were associated with lower non-medical ANC uptake. In Berlin, being a 2nd generation woman or having a low income was also associated with lower uptake.

**Conclusions for Practice:**

Our study suggests that non-medical ANC remains in some part the prerogative of non-migrant, well-educated and economically privileged women. Since differences in non-medical ANC have the potential to create inequalities in terms of birth outcomes and maternal health during pregnancy and post-partum, more efforts are needed to promote the use of non-medical ANC by all population groups.

## Significance

*What is already known on this subject?* Non-medical antenatal care (ANC) contributes to an enhanced pregnancy experience and improved birth outcomes. A recent study showed a lower uptake of non-medical antenatal care among migrants than among women without migration background using unadjusted data. Socioeconomic status has been hypothesized to impact the utilization of non-medical ANC.

*What this study adds?* This study highlights the existence of social inequalities in non-medical ANC use. Our results suggest that there is a strong relationship between the use of non-medical ANC and migration background and educational attainment, with 1st generation migrants and women with low and medium levels of education having a lower uptake of services.

## Introduction

Antenatal care (ANC) refers to the care that a pregnant woman receives throughout her pregnancy in order to maintain maternal and perinatal health (Vetter and Goeckenjan 2013). It can be divided into medical ANC and non-medical ANC.

Medical ANC refers to the series of recommended medical appointments, screenings, and tests, that a woman is advised to book from the first trimester to the end of her pregnancy. Recommendations with regard to the number of points of contacts with the care provider, their intervals, the type of professional delivering the care (general practitioner, gynaecologist, midwife) and the content of each consultation (e.g. blood test, ultrasound) vary across countries. There is a consensus however on the importance of ANC and solid evidence that it can contribute to a decrease in maternal and perinatal morbidity and mortality (Heaman et al. 2013; Raatikainen et al. 2007; Reime et al. 2009). In Germany, the scope of medical ANC is defined at the federal level by the maternity guideline (Gemeinsamer Bundesausschuss (G-BA) 2019). It entails medical appointments, (e.g. early in pregnancy to confirm the pregnancy; post-partum), three ultrasound screenings in weeks 9–12., 19–22 and 29–32, some tests and examinations (e.g. screening on gestational diabetes in week 25–28) and regular monthly (and in the third trimester more frequent) appointments to control the woman’s blood pressure, urine, and the foetus’s development and position. The regular control appointments can be led by a gynaecologist or a midwife, but the other services are to be provided by a gynaecologist.

Most women in Europe begin medical ANC in their first trimester (Euro-Peristat project with SCPE and Eurocat 2013), although some studies have shown that, in England and Italy for example, women of low socioeconomic status and migrants are particularly at risk of not booking, or booking late, their medical ANC appointments (Phillimore 2016; Lauria et al. 2013). By contrast, in Germany, the timely uptake of medical ANC is relatively high across all population groups, including among women with a migration background (Brenne et al. 2015).

Non-medical ANC on the other hand refers to a range of non-medical services available to expectant women during pregnancy aiming at supporting women and prepare them for the birth and the postpartum period (Bundeszentrale für gesundheitliche Aufklärung (BZgA) 2014). In Germany, non-medical ANC they include antenatal classes, breastfeeding classes and pregnancy-specific yoga or gymnastics courses. Antenatal classes are one time weekend classes or weekly evening classes which start around the 25th week of gestation and end around 4 weeks before birth. These courses are usually delivered by a midwife and covered by the health insurance of expectant parents (Bundeszentrale für gesundheitliche Aufklärung (BZgA) 2014). They include information about the pregnancy, birth and the postpartum period (i.e. breastfeeding). They also provide a space for expectant parents to interact with peers and share experiences. Other types of classes (e.g. yoga, gymnastics, pelvic floor exercise) start at any time during pregnancy and are covered under certain conditions by health insurance schemes. They are usually delivered in hospital, birth centres or community centres but can also be on offer in commercial fitness studios. Hospitals and birth centres also offer information evenings and visits with an aim to answer all the questions expectant parents may have with regard to the birth and to alleviate some of the anxiety that may be related to the facilities. Another type of non-medical ANC is choosing to be cared for by a midwife during pregnancy in addition to the medical care provided by the gynaecologist (Gemeinsamer Bundesausschuss (G-BA) 2019)—although this possibility is often hindered by the chronic shortage of midwives and the lack of understanding of their role (Lohmann et al. 2018). Finally, women may make use of pregnancy-specific counselling provided by the city (e.g. services for single mothers, parental leave and parental pay counselling, etc.) or psychological services (covered by the health insurance with referral from a doctor) at any time during their pregnancy. Access to (mostly) free non-medical ANC can be considered as a set of preventative measures aiming at supporting safe and healthy pregnancy and birth experiences. Use of the services is supported by the Federal Center for Health Education (BZgA), an institution within the Ministry of Health whose goal is to prevent health risks and encourage health-promoting lifestyles (Bundeszentrale für gesundheitliche Aufklärung (BZgA), without year).

There is evidence in the literature that backs up the support for non-medical ANC. Studies indeed suggest that various types of non-medical ANC carry benefits for both the women and their babies. For example, Consonni et al. (2010) have shown that an antenatal programme including education sessions, a visit to the birth facilities, physiotherapeutic exercises and interactive sessions to share pregnancy experiences was linked to less maternal anxiety, more vaginal deliveries and shorter newborn hospitalizations (Consonni et al. 2010). Fenwick et al. (2015) highlighted the benefits of midwife-led psychoeducation in addition to normal maternity care with regard to fear of childbirth and distressing post-birth flashbacks (Fenwick et al. 2015). Other studies have also linked antenatal classes to higher frequency of vaginal deliveries (Afshar et al. 2017), higher rate of exclusive breastfeeding (Sehhatie et al. 2019), and decreased likelihood of birth-related PTSD (İsbir et al. 2016). Maintaining physical activity during pregnancy has a range of benefits (Nascimento et al. 2012). In particular, recent studies have pointed out the various benefits of prenatal yoga classes on the physical and psychological health of women in the perinatal period (Mooventhan 2019; Styles et al. 2019).

Unlike for medical ANC, little is known about the uptake of non-medical ANC across different socioeconomic groups in Germany. Considering the out-of-pocket payments required for some of the non-medical ANC services, it is expected that there would be a gradient with regard to socioeconomic status and the consumption of non-medical ANC (Baron et al. 2015). However, the uptake of antenatal care can also be influenced by a range of socio-cultural and migration-related factors, such as knowledge of and expectations toward the health system (Almeida et al. 2014a, b), perception of the appropriateness and quality of ANC (Fabian et al. 2005), and pregnancy-related beliefs and cultural norms, e.g. exercising in pregnancy may not be considered as safe practice (Benza and Liamputtong 2014; Watson et al. 2016). In this context, the role of migration background and socioeconomic status as possible interacting and influencing factors is yet to be explored. In 2017, 23.6% of the population in Germany (19.3 out of 81.7 million persons) had a migration background (i.e. they were either born outside Germany or were born in Germany and have a foreign nationality or have at least one parent who was born outside Germany or has a foreign nationality). The two largest migrant groups were from Turkey and from Eastern European countries (Statistisches Bundesamt (Destatis) 2018).

In a study set in Berlin, Brenne et al. (2015) showed that the uptake of non-medical ANC was lower among migrants than among women without migration background using unadjusted data (Brenne et al. 2015). They, however, did not perform further analyses aiming to identify factors explaining this difference. Building on this work, there is currently an opportunity to explore further those issues within the German context by analysing the combined baseline data of two German cohort studies: the aforementioned Berlin perinatal study (Brenne et al. 2015) and the BaBi birth cohort study based in Bielefeld (Spallek et al. 2017). While both studies are comparable in their designs and intent, they were conducted in environments with different socioeconomic characteristics and have rather different participants’ profiles. The sample in Bielefeld is on average better off and more educated, compared to the Berlin one. This observation holds when comparing across the two samples the groups of women with a migration background. The relatively high level of education among Bielefeld’s participants with and without a migration background introduces more variation to the total sample. By combining the two samples we can reach full breadth in terms of socioeconomic and migration background.

The concomitant availability and the compatibility of these two datasets offer the unique possibility (i) to test the hypothesis that women with different migratory and socioeconomic backgrounds do not use non-medical ANC in the same way and (ii) to investigate how migrant and socioeconomic status interact in influencing the use of these services by looking for the first time at the interaction between those two factors.

## Methods

The analyses combine baseline data from the Berlin perinatal study (2011–2012) and the BaBi study (2013–2016). Baseline data included modules on pregnancy-related health, health care and behaviours and on socioeconomic and migration backgrounds. More details about the aims and designs of those studies can be found elsewhere (Brenne et al. 2015; Spallek et al. 2017). The participants were introduced to the study by researchers in one-to-one conversations during which were answered any questions they may have had. Standardized face-to-face interviews were then conducted. In the BaBi study 1/3 of participants were interviewed as from the 12th weeks of gestation (of which 80% between the 25th week of pregnancy and birth) and 2/3 up to 8 weeks after birth, whereas in the Berlin perinatal study all participants were interviewed in hospital around the time of birth. Women were recruited in hospitals (Berlin and Bielefeld) and gynaecologist/midwife practices (Bielefeld)—sensitivity analyses (not shown) were conducted to check that the different recruitment strategies did not impact the findings of our study. In addition to German, interviews were offered in Arabic, English, French, Kurdish, Polish, Russian, Spanish and Turkish in Berlin (David et al. 2017) and in Polish, Turkish or English in Bielefeld. The choice of the different languages reflected the composition of the migrant population in both cities at the time of recruitment. Eastern European, Turkish and residents from countries of the former Soviet Union were in both cities among the largest groups of residents with a migration background (Amt für Statistik Berlin-Brandenburg 2019; Ministerium fuer Kinder Familie Fluechtlinge und Integration des Landes Nordrhein-Westfalen 2018). Women who were not able to or comfortable answering questions in one of those languages were excluded from the study. In addition, women had to be at least 18 years old at the time of interview. All participants gave their written informed consent prior to their inclusion in the studies. The BaBi study was approved by the ethical committee of the Medical Faculty of Muenster University and the Data Protection Board of Bielefeld University. The Berlin perinatal study was approved by the Ethics Committee of Berlin Charité, Ethics Commission I Charité Mitte (No. EA 1/235/08).

### Description of the Main Variables

The outcome of interest is the use or non-use of, *non-medical antenatal care*, i.e. declaring to have used at any point during the pregnancy any non-medical pregnancy-related services among or beyond those listed in the questionnaire (i.e. “Yes” to at least one of the questions in Table 1). In descriptive analyses we look at the use of *specific services*, e.g. pregnancy exercise classes, antenatal classes, prenatal care by ambulatory midwife, information visits of clinics and birth houses and consultation services for financial, social or psychological concerns. Additionally we analyse factors being associated with the uptake of *antenatal classes* specifically, as literature suggests that these classes are particularly beneficial to the perinatal phase (e.g. Afshar et al. 2017; Consonni et al. 2010).

**Table 1:** Questions used in the cohort studies to capture the use of non-medical ANC

Which of the following services do you use/have you used during this current pregnancy—excluding the medical care provided by a gynaecologist?:
Pregnancy exercise class—yoga, sport, swimming…
Pregnancy care by a midwife
Antenatal class
Information evening or visits of hospitals or birth houses
Social or financial counselling in pregnancy-related counselling services
Psychological counselling
Other service

*Migration background* is determined using the country of birth rather than nationality (Schenk et al. 2006). Women are categorised as follows: first generation (women born abroad from parents born abroad), second generation (women born in Germany from parents both born abroad), third generation (women born in Germany from parents born in Germany, but whose first language is not German) and no migration background. Second and third generation women are grouped together under the “2nd generation” label as only few third generation women were identified. Women born in Germany with only one parent who immigrated are considered as without migration background (Schenk et al. 2006).

*Socioeconomic status* is measured through income and education. *Income* is the self-declared monthly net household income (independent of persons living in the household) and classified in categories: < 900, 900–1500, > 1500–2600, > 2600 Euro. *Education* levels are broken down between low, medium, and high. For each category, the maximum educational attainment is respectively (i) completing high school, (ii) an additional technical/vocational degree or apprenticeship, (iii) a bachelor degree or equivalent and above.

The following factors are also considered as potential confounders: *age* (18–24, 25–29, 30–34, 35 +), and *parity* (primiparous, multiparous).

### Analysis

Descriptive statistics are used to describe the two study samples regarding the aforementioned characteristics. Multivariate logistic regression analyses are performed to study associations between the uptake of non-medical ANC and migration background, educational attainment and income in both study settings. All logistic regression models are adjusted for age and parity. Linear regression analyses are used for collinearity diagnostics (no collinearities were found). Interaction analyses are used to detect effect modifications. Interactions between two terms are tested in the final fully adjusted model if those terms show a statistically significant association with the outcome in the regression model. Marginal probability differences are calculated to reflect the effect of one unit change in the independent variable (e.g. 0 = women without migration background, 1 = 1st generation migrants) on the probability of showing the event of the dependent variable (e.g. using non-medical ANC) (Wright, without year). The significance level is set at p < 0.05. All analyses are performed with SAS 9.4.

## Results

### Description of the Sample

Table 2 presents the main characteristics of the sample comprising participants from the BaBi study and the Berlin perinatal study. It distinguishes between women without migration background, 1st generation migrants and 2nd generation women. For most variables in both settings, the group without migration background differs substantially from the two other groups. Women without migration background are proportionately older, have higher levels of educational attainment and higher incomes. The groups with a migration background are predominantly multiparous.

**Table 2:** Main characteristics of the study samples by migration status (BaBi study and Berlin perinatal study), n = 7044

	BaBi study			Berlin perinatal study		
	Women without migration background	1st generation migrant women	2nd generation women	Women without migration background	1st generation migrant women	2nd generation women
N	468	184	78	3105	2328	881
*Age*						
18–24	26 (5.6%)	23 (12.5%)	8 (10.3%)	522 (16.8%)	471 (20.2%)	219 (32.9%)
25–29	109 (23.3%)	53 (28.8%)	25 (32.1%)	754 (24.3%)	691 (29.7%)	286 (32.5%)
30–34	211 (45.1%)	68 (37.0%)	28 (35.9%)	1017 (32.8%)	648 (27.8%)	191 (21.7%)
35+	122 (26.1%)	40 (21.7%)	17 (21.8%)	812 (26.2%)	518 (22.3%)	114 (12.9%)
*Educational attainment*						
Low	44 (9.4%)	44 (23.9%)	17 (21.8%)	452 (14.6%)	914 (39.3%)	337 (38.3%)
Medium	172 (36.8%)	88 (47.8%)	41 (52.6%)	1615 (52.0%)	937 (40.3%)	484 (54.9%)
High	252 (53.9%)	52 (28.3%)	20 (25.6%)	1038 (33.4%)	477 (20.5%)	60 (6.8%)
*Household net income*						
< 900 €	33 (7.1%)	16 (8.7%)	5 (6.4%)	359 (11.6%)	612 (26.3%)	195 (22.1%)
900–1500 €	34 (7.3%)	19 (10.3%)	12 (15.4%)	705 (22.7%)	962 (41.3%)	369 (41.9%)
> 1500–2600 €	74 (15.8%)	70 (38.0%)	23 (29.5%)	882 (28.4%)	481 (20.7%)	216 (24.5%)
> 2600 €	327 (69.9%)	79 (42.9%)	38 (48.7%)	1159 (37.3%)	273 (11.7%)	101 (11.65%)
*Parity*						
Primiparous^a^	221 (47.2%)	62 (33.7%)	28 (45.9%)	1748 (56.3%)	829 (35.6%)	390 (44.3%)
Multiparous	247 (52.8%)	122 (66.3%)	50 (64.1%)	1357 (43.7%)	1499 (64.4%)	491 (55.7%)

^a^Women who have never born a child before

The majority of study participants in both settings uses non-medical ANC services (see Table 3). With the exception of consultation on parental benefits and social care counselling in Bielefeld, the uptake of all non-medical ANC services is higher among women without migration background as opposed to the two groups with a migration background. The uptake of antenatal classes, pregnancy sports and information visits at birth clinic or birth houses is higher among participants in the BaBi study compared to the Berlin perinatal study.

**Table 3:** Utilization of non-medical ANC among women with and without migration background (BaBi study and Berlin perinatal study)

	BaBi study			Berlin perinatal study		
	Women without migration background (%)	1st generation migrant women (%)	2nd generation women (%)	Women without migration background (%)	1st generation migrant women (%)	2nd generation women (%)
Non-medical ANC, among which^a^	422 (90.2)	129 (70.1)	62 (79.5)	2667 (85.9)	1502 (64.5)	646 (73.3)
Antenatal classes	292 (69.2)	63 (48.8)	34 (54.8)	1146 (43.0)	265 (17.6)	97 (15.0)
Sports/gymnastics, yoga	207 (49.1)	48 (37.2)	19 (30.7)	727 (27.3)	196 (13.1)	71 (11.0)
Prenatal care by ambulatory midwife	269 (63.7)	69 (53.5)	28 (45.2)	1980 (74.3)	654 (43.5)	322 (49.9)
Information evenings and visit of clinics or birth houses	272 (64.5)	73 (56.6)	28 (45.2)	1228 (46.0)	384 (25.6)	136 (21.1)
Consultation on parental benefits and social care counselling	34 (8.1)	13 (10.1)	5 (8.31)	377 (25.4)	279 (18.6)	152 (23.5)
Psychological counselling	9 (2.1)	0^b^	0 ^b^	62 (2.3)	20 (1.3)	9 (1.4)

^a^Percentages for the different types of services below refer to the subsample of women who declared using non-medical ANC. They are therefore higher than would have been expected when referring to the total sample of women included in the analyses

^b^For n < 5 numbers not shown to secure data protection

## Regression analyses

Tables 4 and 5 show the odds ratios and marginal effect differences for the uptake of non-medical ANC and antenatal classes. All marginal effects differ between women with and without migration background. First generation migrants as opposed to women without migration background are less likely to use non-medical ANC and antenatal classes in both study settings (Bielefeld: adjusted OR_BaBinmANC_ 0.40; CI 0.24–0.65 and adjusted OR_BaBiantenatal_class_ 0.42; CI 0.28–0.65; Berlin: (adjusted OR_BerlinnmANC_ 0.33; CI 0.21–0.50 and adjusted OR_Berlinantenatal_class_ 0.37; CI 0.31–0.44). Furthermore, the fact of having attained a low or medium level of education is statistically associated with lower uptake of both non-medical ANC and antenatal classes. In Berlin income plays a role in the uptake of non-medical ANC and antenatal classes: whereas an income below 900€ decreases the chance of using non-medical ANC, earning more than 2600€ increases it (Table 4). A household income below 1500€ is associated with lower uptake of antenatal classes (Table 5). Second generation women are less likely to use antenatal classes (adjusted OR_Berlinantenatal_class_ 0.34; CI 0.27–0.44). In other words, the marginal probability difference of − 0.130 for 2nd generation immigrants in Berlin means that their probability of using antenatal classes is 13.0% points lower than for non-immigrants.

**Table 4:** Influencing factors of using non-medical ANC in the BaBi study and Berlin perinatal study, adjusted for age and parity

	BaBi study				Berlin perinatal study			
	OR	95% CI	p value	Marginal probability difference	OR	95% CI	p value	Marginal probability difference
Migration status (ref: women without migration background)								
1st generation migrant women	0.40	0.24–0.65	0.0002	− 0.099 (− 0.105 to − 0.094)	0.33	0.21–0.50	< 0.0001	− 0.178 (− 0.180 to − 0.178)
2nd generation women	0.71	0.36–1.42	0.3322	− 0.037 (0 − .039 to − 0.035)	1.17	0.50–2.74	0.7117	0.025 ( 0.0.25 to 0.026)
Age (ref: 25–29)								
18–24	0.42	0.17–1.03	0.0507	− 0.094 (− 0.100 to − 0.089)	0.81	0.67–0.97	0.0194	− 0.035 (− 0.035 to − 0.034)
30–34	0.64	0.34–1.17	0.1475	− 0.049 (− 0.052 to − 0.046)	1.04	0.88–1.23	0.0504	0.006 ( 0.006 to 0.006)
35+	0.46	0.24–0.88	0.0194	− 0.084 (− 0.089 to − 0.079)	1.06	0 88–1.28	0.5310	.009 (0.009 to − 0.010)
Educational attainment (ref: high)								
Low	0.15	0.07–0.32	< 0.0001	− 0.204 (− 0.216 to − 0.193)	0.43	0.29–0.63	< 0.0001	− 0.137 (− 0.139 to − 0.136)
Medium	0.22	0.12–0.42	< 0.0001	− 0.161 (− 0.170 to − 0.152)	0.58	0.43–0.78	0.0003	− 0.088 (− 0.089 to − 0.087)
Household net income (ref: (900–1500 €)								
< 900€	1.27	0.49–3.28	0.6267	− 0.013 (− 0.014 to − 0.012)	0.89	0.64–1.24	0.4903	− 0.019 (− 0.019 to − 0.018)
> 1500–2600€	1.43	0.67–3.04	0.3557	− 0.038 (− 0.040 to − 0.036)	1.26	0.95–1.67	0.1107	0.037 ( 0.036 to 0.037)
> 2600€	1.95	0.92–4.15	0.0838	0.033 ( 0.032 to 0.035)	1.77	1.29–2.43	0.0005	0.091 ( 0.090 to 0.092)
Parity (ref = primiparous)								
Multiparous	0.20	0.11–0.38	< 0.0001	− 0.171 (− 0.181 to − 0.161)	0.36	0.31–0.41	< 0.0001	− 0.166 (− 0.167 to − 0.164)
Interaction terms								
		1st gen. migrant women * low education			1.88	1.18–2.99	0.0078	0.101 ( 0.100 to 0.102)
		1st gen. migrant women * medium education			1.76	1.18–2.63	0.0053	0.091 ( 0.090 to 0.092)
		2nd gen. women * low education			0.87	0.36–2.08	0.7479	− 0.023 (− 0.023 to − 0.023)
		2nd gen. women * medium education			0.83	0.37–1.86	0.6428	− 0.031 (− 0.031 to − 0.030)
		1st gen. migrant women * income < 900€			0.78	0.58–1.20	0.2043	− 0.041 (− 0.041 to − 0.040)
		1st gen. migrant women * income > 1500–2600€			0.83	0.58–1.20	0.3274	− 0.030 (− 0.030 to − 0.029)
		1st gen. migrant women * income > 2600€			0.64	0.41–1.02	0.0598	− 0.071 (− 0.072 to − 0.070)
		2nd gen. women * income < 900€			1.16	0.67–1.99	0.6006	0.023 ( 0.023 to 0.024)
		2nd gen. women * income > 1500–2600€			0.42	0.36–0.67	0.0003	− 0.140 (− 0.141 to − 0.139)
		2nd gen. women * income > 2600€			0.24	0.13–0.45	< 0.0001	− 0.227 (− 0.230 to − 0.225)

**Table 5:** Influencing factors of using antenatal classes in the BaBi study and Berlin perinatal study, adjusted for age and parity

	BaBi study				Berlin perinatal study			
	OR	95% CI	p value	Marginal probability difference	OR	95% CI	p value	Marginal probability difference
Migration status (ref: women without migration background)								
1st generation migrant women	0.42	0.28–0.65	< 0.0001	− 0.149 (− 0.153 to − 0.145)	0.37	0.31–0.44	< 0.0001	− 0.123 (− 0.125 to − 0.121)
2nd generation women	0.69	0.39–1.23	0.2056	− 0.065 (− 0.067 to − 0.063)	0.34	0.27–0.44	< 0.0001	− 0.130 (− 0.132 to − 0.128)
Age (ref 25–29)								
18–24	0.19	0.08–0.43	< 0.0001	− 0.289 (− 0.297 to − 0.281)	0.53	0.42–0.66	< 0.0001	− 0.079 (− 0.080 to − 0.077)
30–34	0.91	0.57–1.43	0.6666	− 0.017 (− 0.018 to − 0.017)	1.15	0.95–1.40	0.1476	0.017 ( 0.017 to 0.018)
35+	1.07	0.64–1.79	0.7954	0.012 (0.011 to 0.012)	1.03	0.83–1.28	0.7736	0.004 ( 0.004 to 0.004)
Educational attainment (ref: high)								
Low	0.37	0.20–0.69	0.0018	− 0.174 (− 0.179 to − 0.169)	0.23	0.17–0.30	< 0.0001	− 0.182 (− 0.185 to − 0.179)
Medium	0.53	0.36–0.78	0.0012	− 0.111 (− 0.114 to − 0.108)	0.69	0.58–0.82	< 0.0001	− 0.046 (− 0.046 to − 0.045)
Household net income (ref: (900–1500 €)								
< 900 €	0.75	0.31–1.82	0.5281	− 0.049 (− 0.051 to − 0.048)	0.63	0.49–0.81	0.0004	− 0.056 (− 0.057 to − 0.055)
> 1500–2600 €	1.03	0.51–2.06	0.9370	0.005 ( 0.005 to 0.005)	1.59	1.30–1.94	< 0.0001	0.056 ( 0.055 to 0.057)
> 2600 €	1.22	0.63–2.35	0.5573	0.034 ( 0.033 to 0.035)	1.45	1.17–1.79	0.0006	0.045 (0.044 to 0.046)
Parity (ref = primiparous)								
Multiparous	0.11	0.07–0.16	< 0,0001	− 0.389 (− 0.399 to − 0.378)	0.09	0.08–0.11	< 0.0001	− 0.292 (− 0.297 to − 0.287)

Similar results (not shown) where found with regard to education and migration background when looking at the largest subgroup of women with a migration background in both cohorts, i.e. the women originally from Turkey.

In Berlin, the analysis of interactions shows that 1st generation migrant women with low or medium education have higher odds of using non-medical ANC compared to the reference group (1st generation migrant women with high education; see Table 4). Second generation women with a household net income above 1500€ have lower odds of using non-medical ANC than second generation women with an income of 900–1500€. The interaction terms were significant only for the Berlin perinatal study and with regard to non-medical ANC. Other interactions tested for Bielefeld with regard to non-medical ANC and Berlin and Bielefeld for antenatal classes were all non-significant (interactions not shown).

## Discussion

### Implications of results

This study highlights the existence of social inequalities in non-medical ANC use. Our results suggest that there is a strong relationship between the use of non-medical ANC and migration background and educational attainment, with 1st generation migrants and women with low and medium levels of education having a lower uptake of services. Similar associations between education or migration background and non-medical ANC and antenatal classes were found in other studies, too. Baron et al. (2015) showed women with lower educational attainment to be 4.5 times more likely of not attending antenatal classes (Baron et al. 2015). Fabian et al. (2008) found migrant women to show lower attendance of antenatal classes. The literature suggests that the differences in service use between women with lower or higher educational attainments might be due to differing perceptions of the quality and experiences of the said services. In a Swedish cohort study for example (Fabian et al. 2005), women with low educational level were less likely to find antenatal classes helpful, pointing to the need to improve the format of the classes in order to benefit equally in all population subgroups. Phillimore (2016) identified the following reasons for migrants' poor access to antenatal care: lack of information about classes, being unable to communicate with the staff, and a lack of transport or of affordable transport as main barriers to attendance (Phillimore 2016). Less acculturated women may not seek non-medical ANC because they do not see its value (Fabian et al. 2005), they think they do not need it or they do not feel addressed, as it has been shown to be the case for medical ANC (Almeida et al. 2014a, b; Phillimore 2016).

The association between the fact of being first generation migrant woman and uptake shows how important it is to tend to the needs of those who might not be fully familiar with the German healthcare system and the German culture. The persistence of lower rate of non-medical ANC uptake among first and second generation, well-educated women is also an indication that acculturation may play more of a role than economic integration. Interaction terms which suggests that highly educated first generation migrants may use less non-medical ANC than first generation migrants with low educational attainment also point to the complexity of healthcare-seeking behaviours and the role of other social determinants. The heterogeneity of migration trajectories and the role of migrant communities in the country of reception shape different experiences of migration and integration, through different levels of social support and social isolation. Seeking non mandatory healthcare requires a combination of interest and knowledge of the health system which is acquired not only through contact with medical providers but also through peers. One can hypothesize that first generation migrants with high level of education who, for example, moved to Germany for career opportunity, may be more isolated and less familiar with the health system than other groups of first generation women. It is likely that there is a complex interplay of underlying cultural and/or structural barriers which trigger this association.

In an attempt to better understand the effects of migration background and education, we combined these two key determinants, stratifying our samples into 6 subgroups. In Berlin, where the women with migration background differ from the women without migration background in terms of education, there is a clear trend showing that whatever the education level, women with a migration background are less likely to use non-medical ANC. It also shows that utilization tends to increase with education. In Bielefeld, where there is less variance between educational levels of women with and without migration background, education has a positive effect only for highly educated women with a migration background, while women without a migration background and low education do not differ from women with the same education but a migration background. Those differences call for a more nuanced interpretation of the role of migration status and education and a better understanding of the impact of more socio-economic inequalities across population sub-groups.

Furthermore, our results indicate regional differences with regard to the role of income. In Berlin women with lower income were less likely to use non-medical ANC. Even though many antenatal services are without additional costs, other income-related barriers (e.g. distance between home or workplace and classes, lack of transportation) might contribute to explain these findings in a city of the size of Berlin.

### Strength and limitations

This study is the first to look at the social determinants of non-medical ANC use in Germany. One of its strengths is the variance of participants’ profiles within and across the two studies. The similarities between the two studies and the difference in contexts and population profiles are a unique advantage of our analyses. They allow to have a more diverse sample and to understand better the multi-dimensional determinants of non-medical ANC uptake. We were able for example to take into account in the analyses subgroups such as 1st generation migrants and 2nd generation women and to look at the interaction of migration background and education. Other subgroup analyses with categories based on the country of origin of women with a migration background were limited. We were nevertheless able to perform sensitivity analyses looking at women originally from Turkey which showed results similar to our main analyses.

Finally a couple of limitations must be mentioned with regard to how non-medical ANC data was collected: the uptake of non-medical ANC was assessed retrospectively in most of the women in this study, but a third of BaBi study participants had been interviewed during pregnancy. At the time of interview, they may have not taken part yet in non-medical ANC activities but may have gone to do so by the end of the pregnancy. Furthermore, we did not measure adherence to non-medical ANC. For example, if a woman said she attended antenatal classes, she was not asked how many times, during which trimester, etc. We simply measured if women said they use “some” non-medical ANC.

## Conclusion

Women with a migration background, with low educational attainment and to some extent those with low income (only in the Berlin sample) are all less likely to make use of non-medical ANC services or antenatal classes. Our study suggests that non-medical ANC remains in some part the prerogative of non-migrant, highly-acculturated, well-educated and economically privileged women. In order to improve the uptake of non-medical ANC, it is necessary to identify existing structural and cultural barriers. Since differences in non-medical ANC have the potential to create inequalities in terms of birth outcomes and post-partum health, more efforts are needed to promote and potentially (financially) support the use of non-medical ANC by all population groups.
